# Hepatoprotective Effect of MMP-19 Deficiency in a Mouse Model of Chronic Liver Fibrosis

**DOI:** 10.1371/journal.pone.0046271

**Published:** 2012-10-09

**Authors:** Marketa Jirouskova, Olga Zbodakova, Martin Gregor, Karel Chalupsky, Lenka Sarnova, Marian Hajduch, Jiri Ehrmann, Marie Jirkovska, Radislav Sedlacek

**Affiliations:** 1 Institute of Molecular Genetics of the ASCR, Prague, Czech Republic; 2 Institute of Molecular and Translational Medicine, Faculty of Medicine and Dentistry, Palacky University and University Hospital in Olomouc, Olomouc, Czech Republic; 3 Department of Clinical and Molecular Pathology, Faculty of Medicine and Dentistry, Palacky University and University Hospital in Olomouc, Olomouc, Czech Republic; 4 Institute of Histology and Embryology, First Faculty of Medicine, Charles University in Prague, Prague, Czech Republic; French National Centre for Scientific Research, France

## Abstract

Liver fibrosis is characterized by the deposition and increased turnover of extracellular matrix. This process is controlled by matrix metalloproteinases (MMPs), whose expression and activity dynamically change during injury progression. MMP-19, one of the most widely expressed MMPs, is highly expressed in liver; however, its contribution to liver pathology is unknown. The aim of this study was to elucidate the role of MMP-19 during the development and resolution of fibrosis by comparing the response of MMP-19-deficient (MMP19KO) and wild-type mice upon chronic liver CCl_4_-intoxication. We show that loss of MMP-19 was beneficial during liver injury, as plasma ALT and AST levels, deposition of fibrillar collagen, and phosphorylation of SMAD3, a TGF-ß1 signaling molecule, were all significantly lower in MMP19KO mice. The ameliorated course of the disease in MMP19KO mice likely results from a slower rate of basement membrane destruction and ECM remodeling as the knockout mice maintained significantly higher levels of type IV collagen and lower expression and activation of MMP-2 after 4 weeks of CCl_4_-intoxication. Hastened liver regeneration in MMP19KO mice was associated with slightly higher IGF-1 mRNA expression, slightly increased phosphorylation of Akt kinase, decreased TGF-ß1 mRNA levels and significantly reduced SMAD3 phosphorylation. In addition, primary hepatocytes isolated from MMP19KO mice showed impaired responsiveness towards TGF-ß1 stimulation, resulting in lower expression of Snail1 and vimentin mRNA. Thus, MMP-19-deficiency improves the development of hepatic fibrosis through the diminished replacement of physiological extracellular matrix with fibrotic deposits in the beginning of the injury, leading to subsequent changes in TGF-ß and IGF-1 signaling pathways.

## Introduction

Liver fibrosis and its possible progression toward cirrhosis is a result of a wound healing process by which the liver responds to continuous chronic injury. This pathological process engages many cell types and mediators and results in the excess deposition of extracellular matrix (ECM) proteins, causing a distortion of the normal liver architecture and function that potentially lead to liver failure [1]. During hepatic fibrogenesis, the physiologic ECM that is rich in basement membrane components, predominantly type IV collagen (collagen IV), is degraded and replaced by interstitial matrix fibers, i.e. predominantly collagens I and III [2]. Remodeling of ECM is controlled by matrix metalloproteinases (MMPs) whose expression and activity dynamically change during liver injury and recovery [3]. MMPs and their inhibitors, TIMPs, are thought to regulate matrix turnover, and their imbalance determines the processes of fibrogenesis and fibrolysis [4]. It was shown that toxic liver injury is associated with increased levels of most MMPs while TIMPs were downregulated [3]. In contrast, chronic liver injury is characterized by lower matrix degradation correlating with lower MMP expression while TIMP production is augmented [3].

Although it has been shown that general inhibitors of MMPs, for instance, attenuate hepatic fibrosis [5] or are useful to inhibit acute, and chronic inflammatory or vascular diseases as reviewed in [6], we have an incomplete understanding of the roles the individual MMPs may play during liver injury. MMP-2 was shown to be increased in fibrotic liver [7], [8], and its expression was thought to be pro-fibrotic by its ability to degrade collagen IV [9]. However, recent studies have reported that MMP-2-deficient animals show increased liver fibrosis, due to increased collagen I synthesis [10] and suppressed TIMP-1 upregulation [2], [11]. MMP-13, a collagen I degrading MMP, which is highly increased at the beginning of liver injury and during the recovery period, was shown to contribute to an acceleration of liver fibrosis by mediating initial neutrophil infiltration into the cholestatic liver [12]. MMP-9-deficient mice exhibited moderate protection against early fibrosis [13]. Thus, the question arises, do MMP-9 and 13 account for all the hepatoprotective effects of general MMP inhibitors, or do other MMPs also play important roles?

MMP-19 appears to be widely expressed at the mRNA level [14], [15], however, the expression of the protein seems to be restricted to several cell types and tissues [16]–[19]. MMP-19 is able to cleave components of ECM such as laminin 5γ2 chain, nidogen-1, tenascin C, collagen IV, and aggrecan among others [20]–[23]. The role of MMP-19 appears to be prominent in cell types or in compartments where these substrates as well as MMP-19 are simultaneously available, as was documented in the study showing that MMP-19-deficiency causes an accumulation of tenascin-C in bronchial walls of mice suffering from asthma [24]. MMP-19 was also shown to exhibit an anti-tumor effect as it suppresses tumor angiogenesis and invasion [25], [26]. Of note, it has recently been reported that MMP19 may play a protective role during the development of lung fibrosis [27].

However, although MMP-19 is highly expressed in liver [28], [29], its contribution to the pathologic processes in this organ is completely unknown. In this study, we used MMP-19-deficient mice (MMP19KOs) to establish the role of this MMP in the development and resolution of liver fibrosis in mice using CCl_4_ intoxication. We show that MMP-19-deficiency leads to a reduction in the extent of hepatocellular injury and, thus also to a faster recovery from fibrosis.

## Materials and Methods

### Animal Model of CCl_4_-induced Liver Fibrosis

Generation of MMP19KOs was previously described [30]. Experiments were performed with male MMP19KO homozygotes on C57BL/6NCrl background (backcross 21) and control (wild-type, WT) C57BL/6NCrl male mice derived from the backcross breeding. The high number of backcrosses to C57BL/6N background and stable genetic background is important in mouse experimental studies as documented in other studies [31]. MMP19WT littermate controls were used in some experiments with similar results as C57BL/6NCrl mice. To induce fibrogenesis, mice were injected intraperitoneally with 1 µl/g CCl_4_ (diluted 1∶3 with olive oil) or with the vehicle (olive oil control) twice a week for 4 or 6 weeks. Liver and blood samples were collected 48 hours after the last injection under anesthesia. For more details on sample collection see Materials and Methods S1 section.

### Ethic Statement

Animal care and experiments were approved by the Animal Care Committee of the Institute of Molecular Genetics in compliance with national and institutional guidelines.

### Histological Analysis

Specimens were fixed in 4% buffered paraformaldehyde, embedded in paraffin, sectioned, and stained with hematoxylin and eosin (H&E), Sirius Red [32], or processed for immunohistochemistry to detect Ki67 antigen (details in Materials and Methods S1 section).

### Analysis of Serum Parameters

Alanine aminotransferase (ALT) and aspartate aminotransferase (AST) were assessed from serum using kits from Roche Diagnostics (Prague, Czech Republic). Serum levels of MCP1, IL-6, and KC were assayed by ELISA using antibodies from R&D Systems (Minneapolis, MN).

### Quantitative Reverse-Transcriptase Polymerase Chain Reaction (qRT-PCR)

RNA was isolated from liver samples stored in RNAlater® (Ambion, Austin, TX) using the RNeasy Mini Kit (Qiagen, Hilden, Germany) according to the manufacturer's instructions. RNA from samples from the 6-week experiment was isolated from frozen tissue using TRIreagent (Sigma, St.Louis, MO). See supplementary data for further details. Expression of genes of interest was normalized to GAPDH.

### Hydroxyproline analysis

Liver samples were analyzed on the JASCO HPLC (Essex, UK) system equipped with a SC-5ODS column (200 mm×4 mm; Waters) at 0.8 mL/min and 30°C using 470 nm excitation and 540 nm emission with 0.05 M phosphate buffer (pH 5.0) containing 8% acetonitrile as a mobile phase as described [33]. The peak area of hydroxyproline was normalized to the total amino acid content in lysates and expressed as µM per mg of amino acid (µM/AA).

### Immunoblotting and gelatin zymography

Frozen liver tissues in RIPA buffer or TRI Reagent® (Sigma) were homogenized using TissueLyzer II (Qiagen). Equal amounts of protein were resolved on SDS polyacrylamide gels and transferred to nitrocellulose membranes; proteins were detected using antibodies as detailed in the Supporting Information section.

For zymography, lysates were prepared by homogenization of frozen tissue in PBS containing 1% Triton X-100, 0.5% deoxycholate, and 0.1% SDS and the zymography was performed as described previously [34].

### Primary hepatocyte isolation

Hepatocytes were isolated from WTs and MMP19KOs as described [35]. Hepatocytes were plated on collagen I (0.3 mg/mL; BD, Franklin Lakes, NJ) and cultured in DMEM (Sigma) containing 10% fetal bovine serum (FBS, Invitrogen Life Technologies, Carlsbad, CA), 1% penicillin-streptomycin (PAA, Pasching, Austria) and 0.08 U/mL insulin (Eli Lilly, Indianapolis, IN). After 24 h, cells were serum-starved and then stimulated with recombinant human TGF-ß1 (2.5 ng/mL; R&D Systems). Cells were harvested after 0.5, 1, 6, 24, 48, and 72 h of stimulation for RNA and protein isolation by TRIreagent and processed for qRT-PCR (see Materials and Methods S1 section) and immunoblotting.

## Results

### MMP19KOs exhibit ameliorated injury after chronic CCl_4_-administration

**Figure 1:**
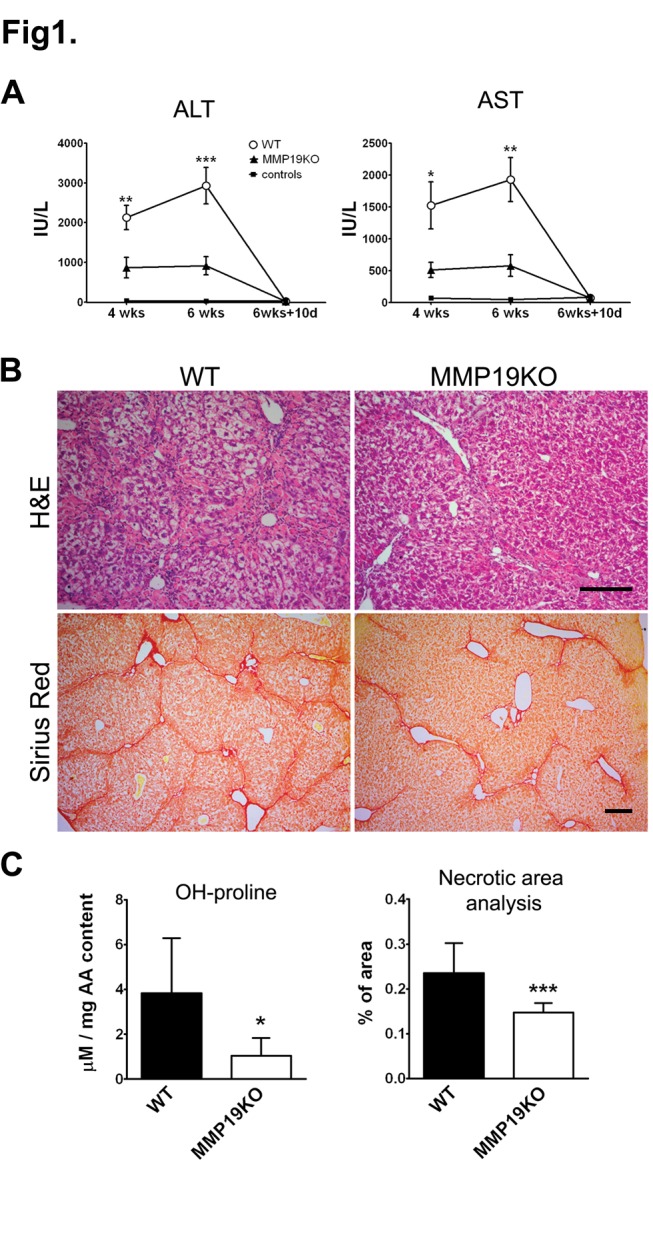
6 week CCl_4_ treatment results in lower liver damage in MMP19KOs compare to WTs. (A) After 4 and 6 week treatment with CCl_4_, serum ALT and AST levels were significantly lower in MMP19KO than in WT mice. WT mice treated with olive oil are shown as control mice. (B) H&E staining showed larger bridging necrosis areas in WTs than in MMP19KOs. Sirius red staining showed more fibrillar collagen deposition in WTs than in MMP19KOs. (C) Liver hydroxyproline (OH-proline) content and image analysis of necrosis areas in H&E stained liver sections showed significantly more extensive damage in WTs than in MMP19KOs. Images were originally taken at 100× (H&E) and 50× (Sirius Red) magnification, scale bars = 200 µm. *p<0.05; **p<0.01; ***p<0.001 MMP19 vs. WT mice; n = 6 olive oil controls, 7 WT and 9 MMP19KO mice.

MMP19KOs exhibit no apparent changes in liver histology [30] and their ALT and AST serum levels are comparable to WTs (Figure S1). To assess the role of MMP-19 in liver fibrosis, we compared liver damage in MMP19KO and WT male mice after 4- or 6-week exposure to CCl_4_. A single dose of CCl_4_ did not result in any significant difference in ALT or AST levels between WT and MMP19KO mice (not shown). However, after 4 weeks of intoxication, while both WT and MMP19KO mice developed serious hepatocellular damage as judged from high serum ALT and AST levels compared to olive oil-treated controls, both markers were significantly higher in WT than in MMP19KO mice (Figure 1A). 6-week CCl_4_-treatment resulted in further increases in ALT and AST levels in WTs but not in MMP19KOs (Figure 1A). Histology revealed typical centrolobular hepatocellular necrosis in both strains (Figure 1B, S2A); however, the necrotic areas were more often bridging in WTs and, after 6 week CCl_4_ treatment, also significantly larger in WTs than in MMP19KOs (Figure 1C). Image analysis of Sirius red staining for fibrillar collagen showed slightly larger fibrotic areas in WTs than in MMP19KOs at both 4 and 6 week time points (Figure S2B). Liver hydroxyproline content correlated with histology, being higher in WTs than in MMP19KOs after 6 weeks of intoxication (Figure 1C). Serum levels of MCP-1 and KC cytokines were only slightly increased in WTs than in MMP19KOs at 4 or 6 weeks of treatment (Table S1), indicating a trend toward decreased inflammatory reaction in MMP19KOs.

Altogether, the observed parameters are consistent with a trend toward decreased hepatocellular injury, fibrosis development, and lower inflammatory reaction in MMP19KOs in response to chronic liver damage.

### MMP19KOs recover faster from fibrotic changes and show higher hepatocyte proliferation during the recovery period

**Figure 2:**
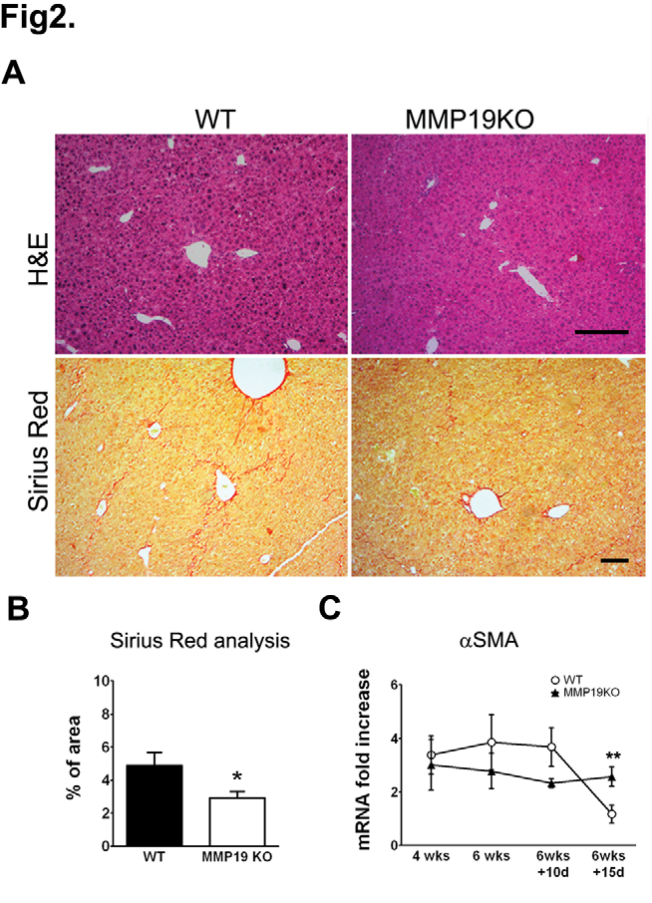
MMP19KOs show faster recovery from fibrosis than WTs. Mice treated with CCl_4_ for 6 weeks were allowed 10 or 15 days for recovery. (A) After 15 days of recovery, significant parenchymal regeneration with less visible necrosis and fibrosis was observed in both WT and MMP19KO livers. (B) Fibrotic area (based on image analysis of Sirius red staining) was significantly lower in MMP19KOs after 15 days of recovery. (D) αSMA mRNA expression was slightly higher in WTs than in MMP19KOs throughout the course of liver damage progression but failed to drop during the recovery. Images were originally taken at 100× (H&E) and 50× (Sirius Red) magnification, scale bars = 200 µm. *p<0.05, MMP19s vs. WTs; 5–9 animals of each strain were analyzed at each time point.

Mice treated for 6 weeks with CCl_4_ were further allowed to recover for 10 or 15 days. Liver function significantly improved after 10 days of recovery, evidenced by ALT and AST levels returning to normal (Figure 1A) in all animals. Even though hepatocytes regenerated in both WTs and MMP19KOs (Figure 2A), histology showed necrotic septa still visible, predominantly in WT livers. Sirius red staining revealed a decrease in fibrillar collagen deposition in both strains with the MMP19KOs showing less extensive fibrotic areas during recovery (Figure 2A, B). Interestingly, there was a further decrease in the extent of fibrotic areas from 10 to 15 days of recovery in MMP19KOs but not in WTs as judged from Sirius red staining (Figure S2B). αSMA mRNA levels, which were slightly lower in MMP19KOs than in WTs during fibrosis development, did not change with fibrosis resolution in MMP19KOs but were significantly decreased in WT animals (Figure 2B). Interestingly, after 10 days of recovery, MMP19 mRNA in WTs significantly increased compared to the period of fibrosis development and then returned to normal levels after 15 days of recovery (Figure S3), thus pointing to a time window during the healing process, in which MMP-19 is engaged in matrix remodeling or removal of fibrotic matrix.

**Figure 3:**
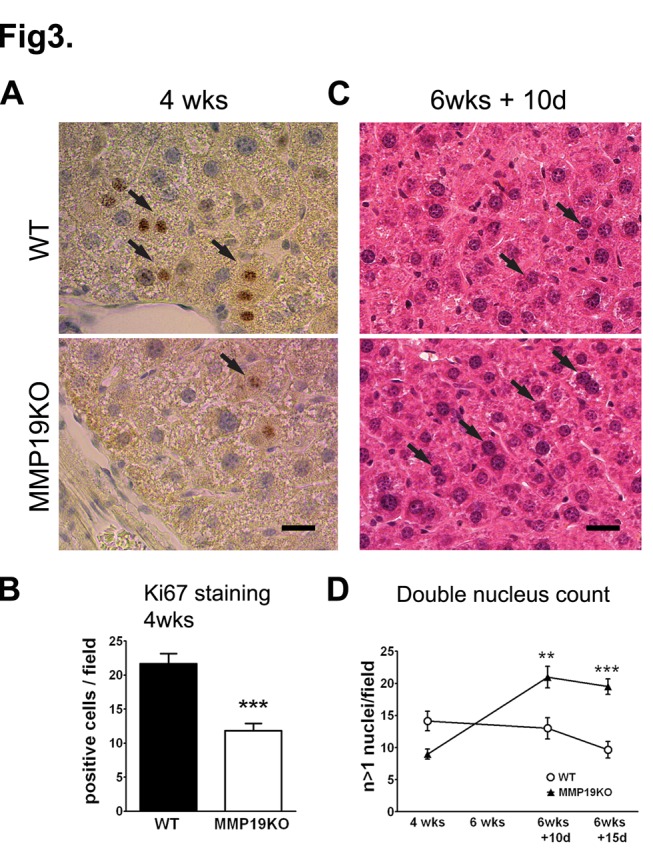
Hepatocyte proliferation in liver during fibrosis resolution is higher in MMP19KOs. (A, B) Ki67 nuclear staining was higher in WTs than in MMP19 KOs after 4 weeks of CCl_4_ treatment. Ki67 positive cells were counted in 20× fields; n = 6 for both WT and MMP19KO. (C, D) Binucleated cells were counted in H&E stained liver sections after 4 weeks of CCl_4_ and in the recovery. (C) Images show representative of counted cells in livers after 10 days of recovery.. (D) During the recovery period the number of double nuclei per field of view was significantly higher in MMP19KO than in WT livers; 4–7 animals of each strain were analyzed at each time point. **p<0.01; ***p<0.001 MMP19s vs. WTs. Scale bars = 1 µm.

As evidenced by the lower ALT and AST levels and histology, hepatocellular damage was substantially lower in MMP19KOs. This led us to investigate the difference in regenerative potency between WT and MMP19KO hepatocytes *in vivo*. Hepatocyte proliferation was studied using Ki67 staining after 4 weeks of CCl_4_ (Figure 3A, B) and by counting binucleated cells (Figure 3C, D). After 4 weeks, both Ki67-staining and binucleated cell counts indicated higher proliferation activity in WT than in MMP19KO hepatocytes, likely reflecting the lower damage in MMP19KO hepatocytes and thus lower demand for reconstitution. However, although we did not observe any Ki67-positive nuclei in either WT or MMP19KO hepatocytes in the recovery period (not shown), binucleated cell counts were significantly higher in MMP19KOs (Figure 3C, D).

### Activity and expression of MMPs during liver damage progression

**Figure 4:**
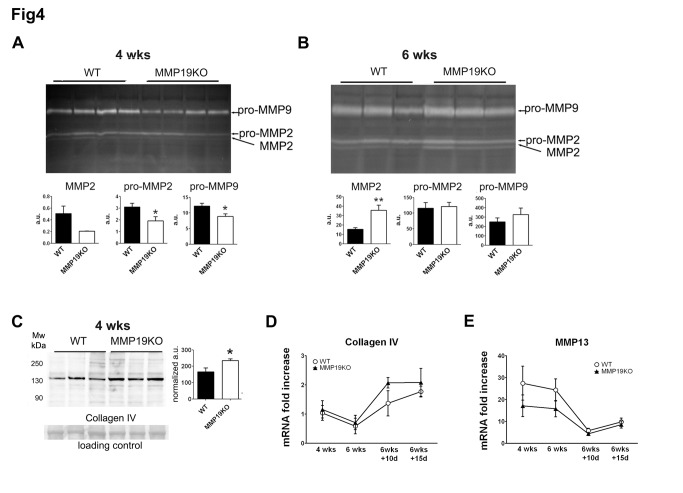
MMPs expression and processing during liver damage progression. (A, B) MMP-2 and MMP-9 expression and activity in liver lysates was analyzed using gelatin zymography. (A) At 4 weeks, WT livers showed higher MMP2 processing and pro-MMP-2 and pro-MMP-9 expression. n = 4 for each strain. (B) At 6 weeks, levels of activated MMP-2 were significantly increased in MMP19KO liver and pro-MMP2 and -MMP9 expression was comparable in WTs and MMP19KOs. n = 6 for each strain. (C) Western blot analysis showed increased collagen IV expression in MMP19KO liver at 4 weeks of CCl_4_. As loading control, the membrane was stained with Ponceau S. n = 10 for each strain. (D) mRNA analysis showed slightly higher collagen IV expression in MMP19 KO than in WT mice. n = 5 animals of each strain analyzed at each time point. (E) qRT-PCR analysis showed slightly reduced expression of MMP13 mRNA levels in MMP19KO mice; 4–9 mice of each strain were analyzed at each time point. *p<0.05, **p<0.01 MMP19KOs vs. WTs.

As the initial destruction of normal ECM during liver injury is controlled by proteases, especially MMPs, we studied the expression and processing of MMP-2 and MMP-9. After 4 weeks of intoxication, both pro-MMP-9 and -MMP-2 were upregulated in both WT and MMPKO mice in comparison to control, olive oil-treated mice (Figure S4). However, MMP19KOs showed significantly lower levels of both pro-MMP-2 and -MMP-9 compared to WTs (Figure 4A). MMP19KOs also exhibited slightly decreased levels of the activated form of MMP-2. In contrast, after 6 weeks of CCl_4_-treatment, the expression of pro-MMP-9 was slightly higher and activated MMP-2 was significantly increased in MMP19KO livers (Figure 4B). When mice recovered from liver injury, pro-MMP-2 and -MMP-9 levels returned to the level of control animals and the activated form of MMP-2 was no longer observed (not shown). Expression of collagen IV, one of the proteins of normal ECM degraded also by MMP-19, correlated well with observed MMP-2 and MMP-9 turnover. We observed significantly more collagen IV in MMP19KO than in WT livers at 4 weeks of CCl_4_-treatment (Figure 4C) and no difference in collagen IV expression at 6 weeks (not shown). Analysis of collagen IV mRNA showed a trend towards slightly higher expression in MMP19KO than in WT livers (Figure 4D). Analysis of MMP-2 mRNA expression showed an increase with CCl_4_-treatment and no differences between the WTs and MMP19KOs after 4 weeks of intoxication (Figure S4). This corresponds well with the zymography data, which showed mainly differences in the regulation of MMP-2 processing. MMP-13 mRNA expression was highly elevated in CCl_4_-treated animals with a trend toward higher levels in WTs than in MMP19KOs (Figure 4E). During the recovery phase, MMP13 expression decreased in both WTs and MMP19KOs.

Altogether, these data show that MMP-19 deficiency appears to impact the proteolytic network of MMPs during the development and recovery of liver fibrosis, reducing their potential to proteolyze ECM, especially in the initial phase of injury when components of the basement membrane are degraded.

### Decreased TGF-ß and increased IGF-1-mediated signaling during fibrogenesis and recovery in MMP19KOs

**Figure 5:**
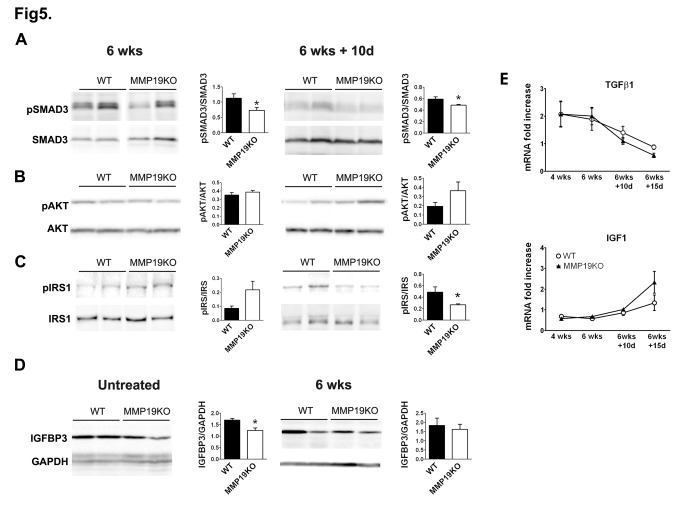
Analysis of TGF-ß- and IGF-1-mediated signaling pathway mediators during hepatic fibrosis and its resolution. (A–C) SMAD3 (A), Akt kinase (B) and IRS1 (C) phosphorylation was analyzed by immunoblotting liver lysates of treated and control animals. (D) IGFBP3 protein expression was significantly lower in untreated MMP19KO than in WT livers. (E) qRT-PCR analysis showed slightly higher IGF-1 and slightly lower TGF-ß1 mRNA levels in MMP19KO livers than in WT livers during fibrosis resolution. *p<0.05, MMP19s vs. WTs. 4–7 mice from each strain were analyzed at each time point. In some instances, noncontiguous lanes from one Western blot are shown.

To reveal signaling pathways which are affected by MMP-19 deficiency, we studied the activation of TGF-ß and IGF-1 pathway components. The phosphorylation of SMAD3, a TGF-ß-signaling mediator, was significantly decreased after 6 weeks of CCl_4_-treatment and in the recovery period in MMP19KO livers (Figure 5A), suggesting that the TGF-ß-mediated pro-fibrotic response was lower in MMP19KO livers. Further analysis of TGF-ß1 mRNA showed a trend toward slightly higher levels of this profibrotic factor in WT livers during the recovery period, further supporting our findings (Figure 5E).

Phosphorylation of IRS1 and Akt kinase was analyzed to obtain insight into IGF-mediated, anti-apoptotic signaling. Immunoblotting analysis revealed slightly increased phosphorylation of Akt in MMP19KOs in the recovery period (Figure 5B), showing a higher activity of this anti-apoptotic signal in the MMP19KOs. Phosphorylation of IRS1 was slightly higher in MMP19KO livers at 6 weeks of CCl_4_-treatment although this reversed and after 10 days of recovery, pIRS1 was significantly lower in MMP19KO than in WT livers (Figure 5C).

Analysis of IGFBP3, an MMP19 substrate involved in IGF-mediated signaling, showed significantly decreased protein levels in untreated MMP19KO livers compared to untreated WT livers (Figure 5D). This difference was smaller after 4 weeks of intoxication (not shown) and the levels of IGFBP3 at 6 weeks were almost identical between WTs and MMP19KOs (Figure 5D). As previously described [34], IGFBP3 processing was significantly diminished in MMP19KO livers compared to WTs (Figure S5). IGFBP3 protein expression was similar between MMP19KO and WT livers during the recovery phase, likely reflecting the increase in IGFBP3 expression as IGFBP3 mRNA levels increased during the recovery phase by 4 fold in both MMP19KO and WT livers compared to the mRNA expression during the intoxication phase (Figure S5). IGF-1 mRNA levels also showed a trend toward being higher in MMP19KOs compared to WTs during the later recovery period (Figure 5E). Thus, these data suggest that MMP-19-deficiency results in lower TGF-ß-mediated hepatocyte damaging signaling and higher IGF-1-mediated, anti-apoptotic and regenerative activity.

### Primary MMP-19-deficient hepatocytes show decreased reaction to TGFß-1 treatment ex vivo

**Figure 6:**
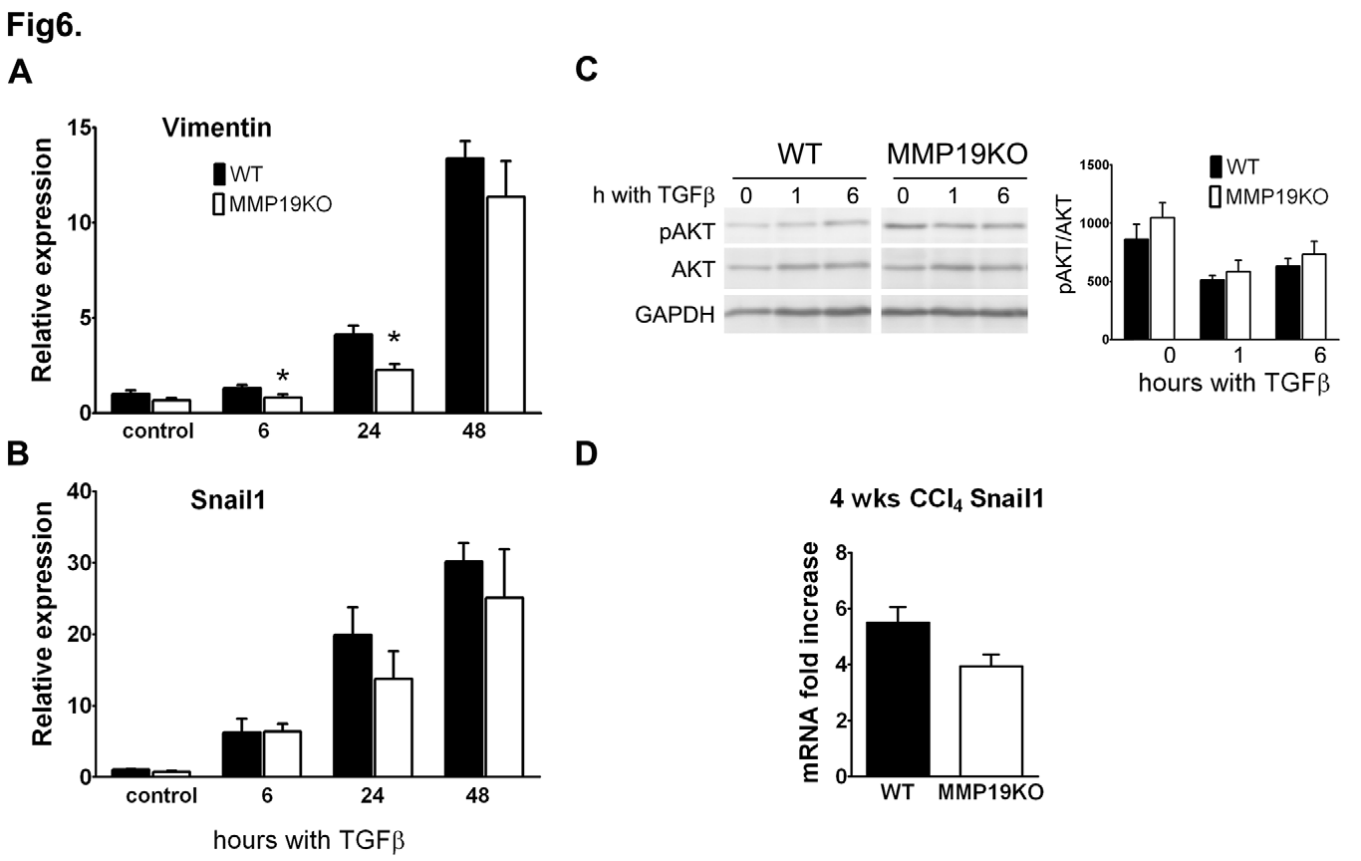
Isolated hepatocytes from MMP19KO livers show impaired responsiveness to treatment with TGF-ß1. Primary hepatocytes were isolated from WT and MMP19KO livers, seeded on collagen I and stimulated with TGF-ß1. (A, B) mRNA levels of vimentin and Snail1 were measured in control, untreated hepatocytes, and hepatocytes stimulated with TGF-ß1 for 6, 24, or 48 h. (C) Phosphorylation of Akt was slightly increased in MMP19KO compared to WT hepatocytes. Noncontiguous lanes from one Western blot are shown. n = 5 different hepatocyte isolations from both WTs and MMP19KOs. (D) mRNA levels of Snail1 in livers from animals treated with CCl_4_ for 4 weeks showed slightly lower expression of Snail1 in MMP19KOs than in WTs. 4–7 mice from each strain were analyzed for each time point. *p<0.05 MMP19KOs vs. WTs.

As CCl_4_-intoxication targets primarily hepatocytes, we used primary hepatocytes to further understand the protective mechanisms in MMP19KO livers. We first studied the effect of CCl_4_ treatment on hepatocytes seeded on collagen I and observed no differences in ALT and AST levels between WT and MMP19KO cells (not shown). Further, the hepatocytes susceptibility to TGF-ß1 stimulation was tested. We measured mRNA expression levels of vimentin and Snail1 in both WT and MMP19KO hepatocytes after TGF-ß1 treatment and studied phosphorylation of the Akt signaling molecule. Vimentin and Snail 1 mRNAs were expressed at low levels in untreated hepatocytes (Figure 6A, B). TGF-ß1 treatment resulted in an increase in their mRNA levels, although this induction was always lower in MMP19KO compared to WT cells. Similar to our observations in vivo, we also observed a trend toward slightly increased phosphorylation of Akt in MMP19KO than in WT hepatocytes, both untreated and treated with TGF-ß1 (Figure 6C). Based on these observations, we also analyzed mRNA expression of Snail1 in liver from treated animals. This analysis also showed slightly lower expression of Snail1 in MMP19KOs than in WTs after 4 weeks of CCl_4_ treatment (Figure 6D). Altogether, these results indicate a reduced susceptibility of MMP19KO hepatocytes to TGF-ß1 stimulation and are consistent with our observation of lower TGF-ß-mediated changes in MMP19KO livers *in vivo*.

## Discussion

In the present study, MMP-19-deficient mice showed resistance to chronic liver injury caused by CCl_4_ intoxication and recovered faster than WT animals upon removal of the toxin. The protective effect of MMP19 deficiency was primarily a result of milder hepatocellular damage, as plasma ALT and AST levels were significantly lower in the knockout animals throughout the CCl_4_ treatment. We also observed decreased liver hydroxyproline content and fibrillar collagen deposition in MMP19KOs throughout disease progression and recovery together with lower phosphorylation of SMAD3, and slightly increased levels of Akt and IRS1 phosphorylation during injury development, suggesting the involvement of TGF-ß- and IGF-1-mediated signaling.

Degradation of normal liver ECM contributes to the pathogenesis of liver fibrosis, particularly in the early stages of injury [36].The ameliorated disease development in MMP19KOs likely originates from slower kinetics of basement membrane destruction and ECM remodeling, as MMP-19 is known to process a number of basement membrane proteins such laminin 2γ chain, tenascin C, collagen IV, and nidogen-1, which all are abundant components of normal liver ECM [20], [22], [23]. After 4 weeks of intoxication, we observed higher collagen IV protein levels in MMP19KO livers after 4 weeks of intoxication, suggesting decreased degradation of this protein. This observation also correlated with the expression and activity of both gelatinases. During the earlier phase of injury, the expression of MMP-2 and MMP-9 and processing of MMP-2 was weaker in MMP19KOs. This lower expression and processing of gelatinase, in turn, likely contributes also to lower processing of collagen IV in MMP19KO livers as MMP-2 efficiently cleaves this type of collagen [37]. After 6 weeks of injury, the expression of both gelatinases was comparable between MMP19KOs and WTs; however, the proportion of activated form of MMP-2 was significantly higher in MMP19KOs. This finding might be pointing to the activity of an additional protease, which converts pro-MMP-2 into the active form. Such a protease might be MMP-14 (MT1-MMP, a membrane type-1 MMP) that together with TIMP-2 activates MMP-2, as all these components are expressed in injured liver [38], [39]. Moreover, MT1-MMP can be upregulated by IGF-1 [40] whose signaling we also observed to be higher in MMP19KOs. The role of MT1-MMP in liver pathology has not been established yet, however, its direct proteolytic activity towards ECM and the activation of MMP2 in the initial phase of liver damage could accelerate the destruction of normal ECM-structure in the liver. Proteolytic activity of MT1-MMP towards collagen I could be beneficial for the resolution of fibrosis [41]. Moreover, MMP-2 appears to also have a protective role in mouse models of liver fibrosis by inhibiting collagen I synthesis and suppressing TIMP-1 upregulation [11] which could thus contribute to lower fibrosis in MMP19KOs. Interestingly, the MMP13 mRNA induction was impaired in MMP19KOs during the initial phase of injury, returning to WT levels during recovery. As MMP-13 was reported to affect acceleration of fibrogenesis in cholestatic livers by mediating the initial inflammation [12], it is likely that downregulation of this interstitial collagenase might be involved in the decreased development of injury in MMP19KOs. This is also in agreement with our observation of increased levels of IGF-1-mediated signaling as IGF-1 was reported to downregulate MMP13 expression [42]. These data suggest that MMP-19 contributes to liver injury, not only by direct processing of components of normal liver ECM, but also by affecting expression and activity of other MMPs probably via the IGF-1 signaling pathway.

Liver injury leads to massive hepatocyte necrosis and hepatic stellate cell activation and is accompanied by decrease in IGF-1 serum levels and induction of TGF-ß. TGF-ß-mediated signaling is pro-apoptotic [43], inhibits hepatocyte proliferation [44], and it was shown that interruption of TGF-ß signaling results in diminished liver fibrosis [45], [46]. IGF-1 reduces formation of fibrosis and enhances liver regeneration [47]. Our data clearly show tendency toward lower TGF-ß-mediated signaling in MMP19KO animals, as we observed less SMAD3 phosphorylation at the end of chronic injury and during the recovery period. As MMP-2 and MMP-9 are both able to activate latent TGF-ß1 [48], lower amounts of active gelatinases in MMP19KOs in the earlier phases of liver injury might also contribute to inhibition of TGF-ß-signaling. Along the same line, phosphorylation of Akt showed an increasing trend in MMP19KO livers during the recovery period, suggesting higher anti-apoptotic signaling either via IGF-1 or another pathway. The Akt pathway also controls a specific cell division program that leads to generation of binucleated tetraploid liver cells and inhibition of Akt was shown to reduce numbers of binucleated cells [49]. Indeed, higher frequencies of binucleated cells in MMP19KO mice in the absence of cellular proliferation markers correlated with increased levels of pAkt. Moreover, there was also a clear tendency towards higher activation of IRS1 during the progression of liver injury, further supporting the possibility that IGF-1 signaling may impact the better outcome in the injury in MMP19KOs. Also, as the hepatocytes are the main source of IGF-1 in liver [50], it is obvious that lower hepatocellular damage in the case of MMP19KO animals by itself may contribute to better regeneration of the disease in these animals. Interestingly, we observed reduced phosphorylation of IRS1 in MMP19KO animals during the recovery period indicating possible deregulation of IGF-1 signaling pathway or other defect in the MMP19KO animals during the recovery. This situation might be reflecting the increased MMP-19 mRNA level in WT animals during recovery suggesting thus an additional role for MMP19 within the recovery period when it is necessary to degrade fibrotic matrix and/or increase the IGF-1 mediated anti-apoptotic signaling. It is plausible, that the increased levels of IGF-1 mRNA levels during the recovery period in MMP19KO animals are part of compensatory mechanism for the impaired IGFBP3 processing and expression, allowing thus for higher levels of bioavailable IGF-1, as there has to be a very stringent regulation of both of these proteins in the liver [50], [51].

There is an increasing evidence that hepatocytes could play a crucial role in orchestrating the TGF-ß1-mediated pro-fibrotic response to chronic exposure to hepatotoxic agents [52]. Our observation that primary hepatocytes isolated from MMP19KO livers express lower levels of vimentin and Snail1 (i.e. genes directly linked to fibrosis progression) mRNA in response to TGF-ß1 stimulation suggests that the MMP19KO hepatocytes are less susceptible to TGF-ß1. This is in agreement with the observation of tendency toward higher phosphorylation of Akt in the MMP19KO hepatocytes as Akt causes resistance to TGF-ß1-induced apoptosis [53]. Consequently, activation of the profibrotic programs in MMP19KO hepatocytes, including those controlled by Snail1 [52], is delayed in comparison to WT cells. Provided that down-regulation of Snail1 in hepatocytes leads to slower fibroproliferative disease progression [52], it seems plausible that the lower activation of Snail1 in MMP-19-deficient hepatocytes not only reflects the observed situation *in vivo* but might also represent a plausible mechanism mediating hepatoprotective effect of MMP-19-deficiency.

Thus we showed that MMP-19 plays an essential role in the development of hepatic damage by toxins and subsequent fibrosis as it affect the destruction and remodeling of hepatic basement membrane components, which in turn influence TGF-ß and IGF-1 signaling pathways and leads to differential expression and activation of other MMPs. As MMP-19 negatively impacts the initial phase of liver injury and may play an additional role during the fibrosis recovery, it may serve as a valuable target for pharmacologic treatment of liver pathologies.

## Supporting Information

Materials and Methods S1 **Detailed Materials and Methods.**
(DOC)

Figure S1 **No differences in ALT, AST, ALP, and bilirubin serum levels in untreated and olive oil-treated control WT or MMP19KO mice were observed.** Control mice were treated with olive oil twice a week for 6 weeks. n = 4 for both strains.
(JPG)

Figure S2 **CCl_4_ intoxication leads to ameliorated liver damage in MMP19KOs.** (A) After 4 weeks of CCl_4_ intoxication, H&E staining showed centrolobular hepatocyte damage more serious in WTs than in MMP19KOs. Sirius red staining revealed larger fibrotic areas in WTs than in MMP19KOs. Images were originally obtained at 100× (H&E) and 50× (Sirius red) magnification, scale bars = 200 µm. (B) Fibrosis, as measured by image analysis of Sirius red staining, was slightly larger in WT than in MMP19KO mice, 5–9 animals of each strain were analyzed at each time point.
(JPG)

Figure S3 **MMP19 mRNA expression during fibrosis development and resolution in WT animals, n = 5.**
(JPG)

Figure S4 **MMP19KO and WT livers show no difference in pro-MMP2 and -MMP9 expression in olive oil-treated control animals.** (A,B) Gelatinase zymography showed no difference in pro-MMP2 and -MMP9 expression in animals treated with olive oil and no activated MMP2 form expression. (C) No differences were observed in MMP2 mRNA expression after 4 weeks of CCl_4_ treatment between WT and MMP19KO livers; n = 5 olive oil controls, 8 WT and 7 MMP19KO mice.
(JPG)

Figure S5 **As previously described, IGFBP3 degradation is lower in MMP19KO livers.** (A, B) IGFBP3 degradation products were lower in MMP19KO livers after 4 and 6 weeks of CCl_4_ treatment. (C) During the recovery period, the IGFBP3 mRNA increased 4-fold in both WT and MMP19KO mice. 4–7 mice from each strain were analyzed at each time point.
(JPG)

Table S1 **MCP1 and KC levels in MMP19KO and WT mice after 4 or 6-week treatment with CCl_4_ were measured in serum using an Elisa kit.**
(DOC)
