# Supplementary material for: Hepatoprotective Effect of MMP-19 Deficiency in a Mouse Model of Chronic Liver Fibrosis
Source: PLoS One. 2012 Oct 9;7(10):e46271. doi: 10.1371/journal.pone.0046271 (PMC3467204; doi:10.1371/journal.pone.0046271)
Supplement: Materials and Methods S1 — Detailed Materials and Methods. (DOC) [file pone.0046271.s001.doc]

**Detailed Materials and Methods:**

***Animal Model of CCl_4_-induced Liver Fibrosis – Sample Collection.***

Liver and blood samples were collected 48 h after the last injection. To study fibrosis resolution, mice were allowed to recover for 10 or 15 days after the last injection. To collect the samples, mice were anesthetized using mixture of Zoletil (Virbac Carros, France) and Rometar (Bioveta, Ivanovice, Czech Republic), blood was collected from retrobulbar plexus and then livers were removed. Samples of the left lateral lobe were collected.

***Histological Analysis.***

Specimens were fixed in 4% buffered paraformaldehyde for 24h, then embedded in paraffin , sectioned, and stained with hematoxylin and eosin (H&E), Sirius Red , or processed for immunohistochemistry. Hepatocyte proliferation was detected by staining with anti-Ki67 (1:50 dilution; Dako, Glostrup, Denmark) antibody and further detected using the DAB Elite kit (DAKO) according to the manufacturer's instructions. The number of positively stained hepatocytes was calculated in four × 20 fields for each sample and expressed as positive cells per field. Images of entire liver sections stained with H&E or Sirius Red were acquired at 2.5x magnification and then analyzed using an open-source ImageJ software (<http://rsb.info.nih.gov/ij/>) to quantify the area of necrosis or fibrosis in each liver section. To quantify nuclear polyploidy, number of cells containing polyploid nuclei was calculated in four x 20 fields of each H&E stained section and expressed as number of positive cells per field.

***Analysis of Serum Parameters.***

Blood from CCl_4_-treated or control animals was allowed to clot for 2.5h at 37^o^C. The clot was removed, remnants centrifuged at 14,000x g for 20 min at 4^o^C, and supernatant stored at -20^o^C for further analysis. Alanine aminotransferase (ALT) and aspartate aminotransferase (AST) were assessed using kits from Roche Diagnostics (Prague, Czech Republic). Serum levels of MCP1, IL6, and KC were assayed by ELISA using Duo set antibodies from R&D Systems (Minneapolis, MN, USA).

***Quantitative Reverse-Transcriptase Polymerase Chain Reaction (qRT-PCR).***

## RNA was isolated from liver samples stored in RNAlater® (Ambion, Austin, TX, USA) using RNeasy Mini Kit (Qiagen, Hilden, Germany) according to the manufacturer’s instructions. RNA from samples from 6-week experiment was isolated from frozen tissue using TRIreagent (Sigma, St.Louis, MO, USA). **500 ng** of total RNA was reverse transcribed at 42°C using 1 µl M-MLV reverse transcriptase (Promega, Madison, WI, USA) and Oligo(dT) at a final concentration of 1.75 µM. cDNA was diluted 1:4 with water and 3 µl were used for real-time PCR performed with SYBR® Green JumpStart™ *Taq* ReadyMix™ (Sigma, St.Louis, MO, USA). The cycling conditions were as follows: denaturation (95°C for 2 min), 45 cycles of amplification (95°C for 15 s, 60°C for 20 s, 72°C for 20 s) and product melting (up to 95°C). Reactions were performed in duplicates on the LightCycler® 480 (Roche, Basel, Switzerland). Expression of genes of interest was normalized to GAPDH.

***Hydroxyproline analysis***

The liver samples were analyzed on the JASCO HPLC (Essex, UK) system equipped with a SC-5ODS column (200 mm × 4 mm; Waters, USA) at 0.8 ml/min and 30^o^C using 470 nm excitation and 540 nm emission with the phosphate buffer 0.05M (pH 5.0) containing 8% acetonitrile as a mobile phase as described . Liver samples (~50 mg, wet weight) were hydrolyzed for 22h at 108^o^C in evacuated flame-sealed Pyrex tubes containing 6 N HCl with 1% phenol. Samples were dried in vacuum and pellets were then resuspended in 300 μl of 50 mM borate buffer containing 20 mM EDTA-2Na (pH 9.0). The derivative procedure was performed with heating the samples for 10 min at 60 ^o^C as described . The peak area of hydroxyproline was normalized to the total amino acid content in lysates and expressed as μM per mg of amino acid (μM/AA).

***Immunoblotting and gelatine zymography***

Frozen liver tissue in RIPA buffer (25 mM Tris , 150 mM NaCl, 10 mM EDTA, 1% Triton X-100, 1% DOC, 0.1% SDS, Complete® inhibitors (Roche), 1 mM ortho vanadate, 2.5 mM sodium pyrophosphate, 1 mM β-glycerolphosphate) was homogenized using TissueLyzer II (Qiagen), cooled 30 min on ice, and clarified at 13 000 x g; tissues were homogenized in TRI Reagent® (Sigma, St.Louis, MO, USA). Protein concentration was determined using BCA assay (Pierce, Rockford, IL, USA); equal amounts were loaded on SDS polyacrylamide gels. After transfer to nitrocellulose membrane, proteins were detected using antibodies directed against pSMAD3, SMAD3 (Abcam, Cambridge, UK; ab51451; ab28379), pAkt and Akt (Cell Signaling Technology, Cell Signaling Technology, Beverly, MA, USA,), pIRS1 and IRS1 (Abcam, Cambridge, UK; ab53038, ab52167), insulin-like growth factor-binding protein 3 (IGFBP3) (R&D Systems; MAB775) or collagen IV (AbD Serotec, Oxford, UK; 2150-1470). GAPDH was used as normalization control (Sigma, G9545).

For zymography, lysates were prepared by homogenization of frozen tissue in PBS containing 1% Triton X-100, 0.5% DOC and 0.1% SDS buffer and the zymography was performed as described previously ). For visualization, gels were stained with PageBlue™ Protein Staining Solution (Fermentas, Burlington, Canada).

***Primary hepatocyte isolation***

Hepatocytes were isolated from WT and MMP19KO mice as described by Moldéus et al. (672656). Isolated hepatocytes were plated on 6-well culture plates (Corning Incorporated, Corning, NY, USA) coated with coll I (0.3 mg/ml, BD, Franklin Lakes, NJ, USA). After 2 hours, medium was changed and cells were further cultured in Dulbecco's Modified Eagle Medium (DMEM, Sigma, St.Louis, MO, USA) supplemented with 10% fetal bovine serum (FBS, Invitrogen Life Technologies, Carlsbad, CA, USA), 1% PenStrep (PAA, Pasching, Austria) and 0,08U/ml insulin (Eli Lilly, Indianapolis, IN, USA). After 24 h of cultivation at 37 °C under 5% CO_2_, the culture medium was replaced with serum-free DMEM. Recombinant human TGF-β1 (R & D Systems, Minneapolis, MN) was added after 1 h starvation at 2.5 ng/ml. Representative images of cells treated with TGF-β1 and control untreated cells were acquired on microscope (Leica DMI6000, Leica) equipped with phase-contrast and epillumination optics. Cells were harvested at 0.5, 1, 6, 24, 48, and 72 h after stimulation for RNA isolation by TRIreagent (Sigma, St.Louis, MO, USA) and processed for qRT-PCR as described above.
