# Supplementary figures and images for: Hepatoprotective Effect of MMP-19 Deficiency in a Mouse Model of Chronic Liver Fibrosis
Source: PLoS One. 2012 Oct 9;7(10):e46271. doi: 10.1371/journal.pone.0046271 (PMC3467204; doi:10.1371/journal.pone.0046271)

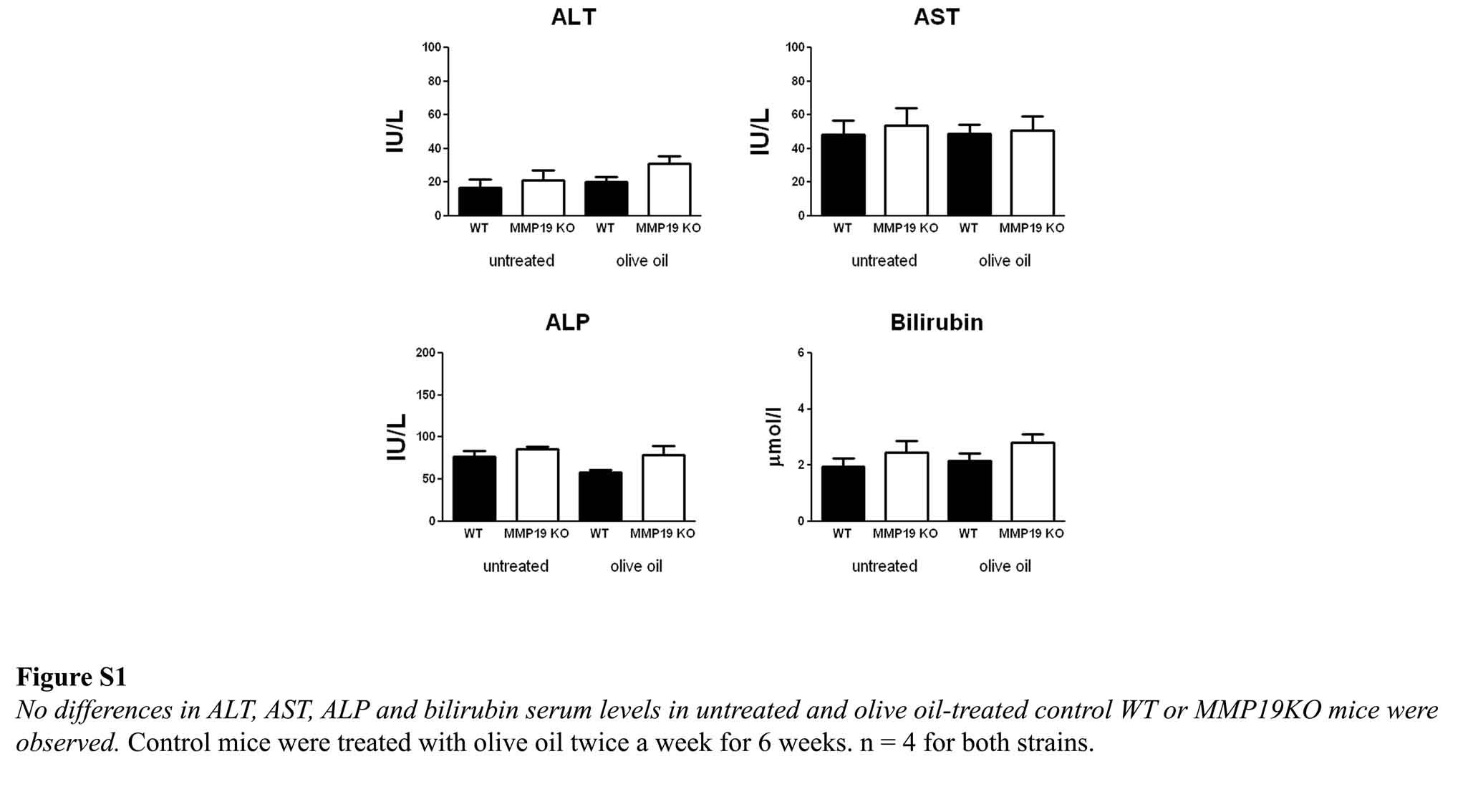

Supplement: Figure S1 — No differences in ALT, AST, ALP, and bilirubin serum levels in untreated and olive oil-treated control WT or MMP19KO mice were observed. Control mice were treated with olive oil twice a week for 6 weeks. n = 4 for both strains. (JPG) [file pone.0046271.s002.jpg]

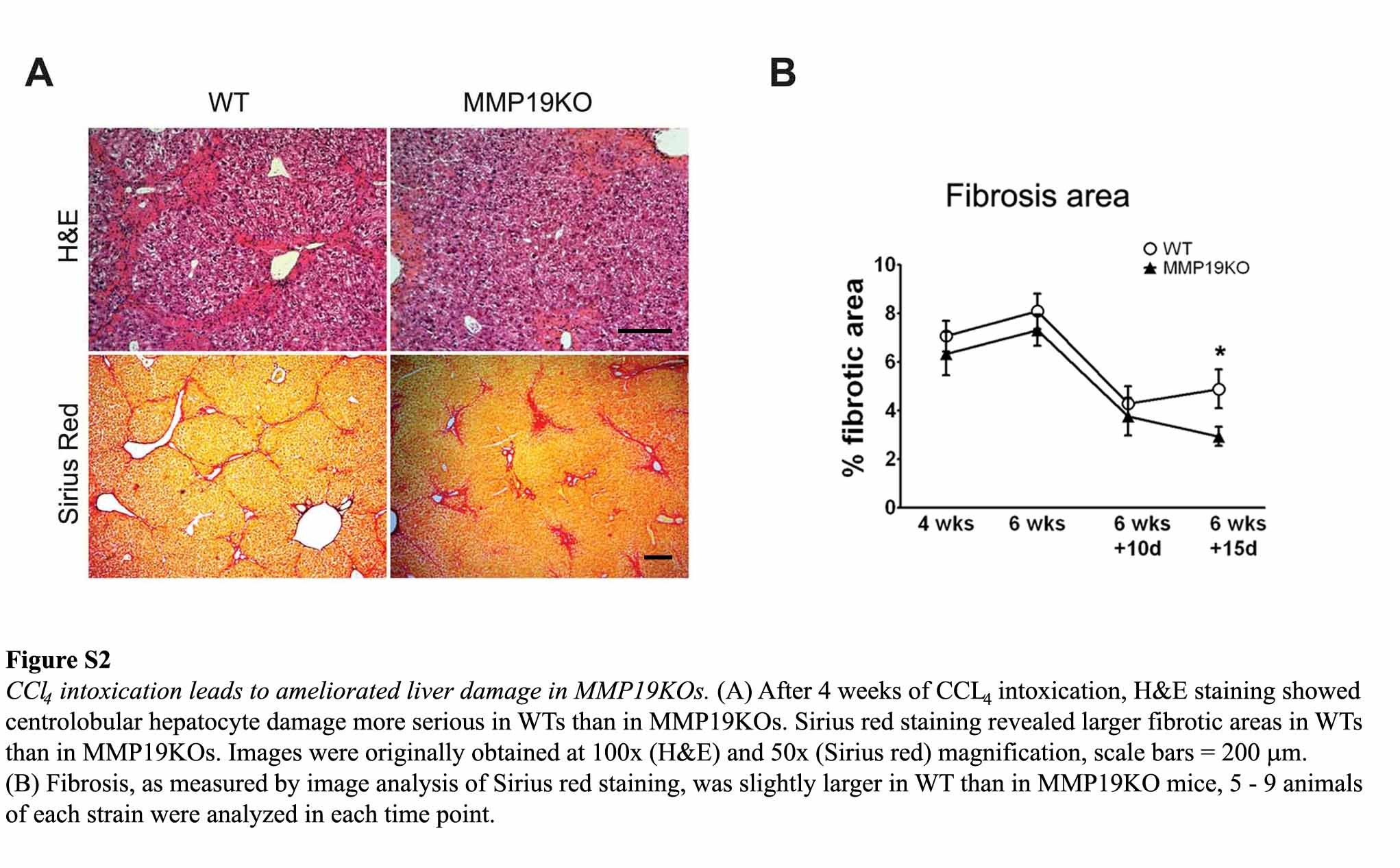

Supplement: Figure S2 — CCl4 intoxication leads to ameliorated liver damage in MMP19KOs. (A) After 4 weeks of CCl4 intoxication, H&E staining showed centrolobular hepatocyte damage more serious in WTs than in MMP19KOs. Sirius red staining revealed larger fibrotic areas in WTs than in MMP19KOs. Images were originally obtained at 100× (H&E) and 50× (Sirius red) magnification, scale bars = 200 µm. (B) Fibrosis, as measured by image analysis of Sirius red staining, was slightly larger in WT than in MMP19KO mice, 5–9 animals of each strain were analyzed at each time point. (JPG) [file pone.0046271.s003.jpg]

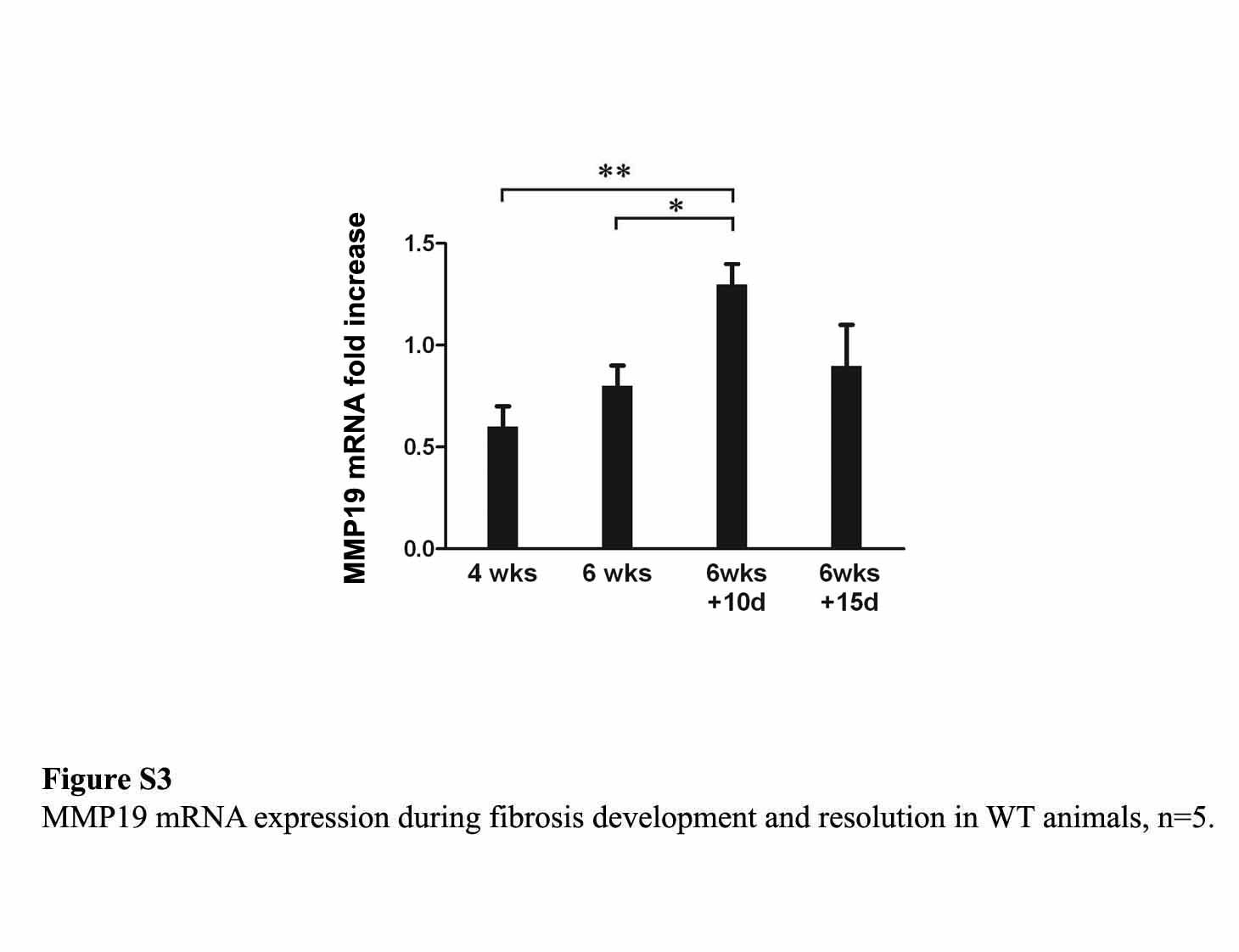

Supplement: Figure S3 — MMP19 mRNA expression during fibrosis development and resolution in WT animals, n = 5. (JPG) [file pone.0046271.s004.jpg]

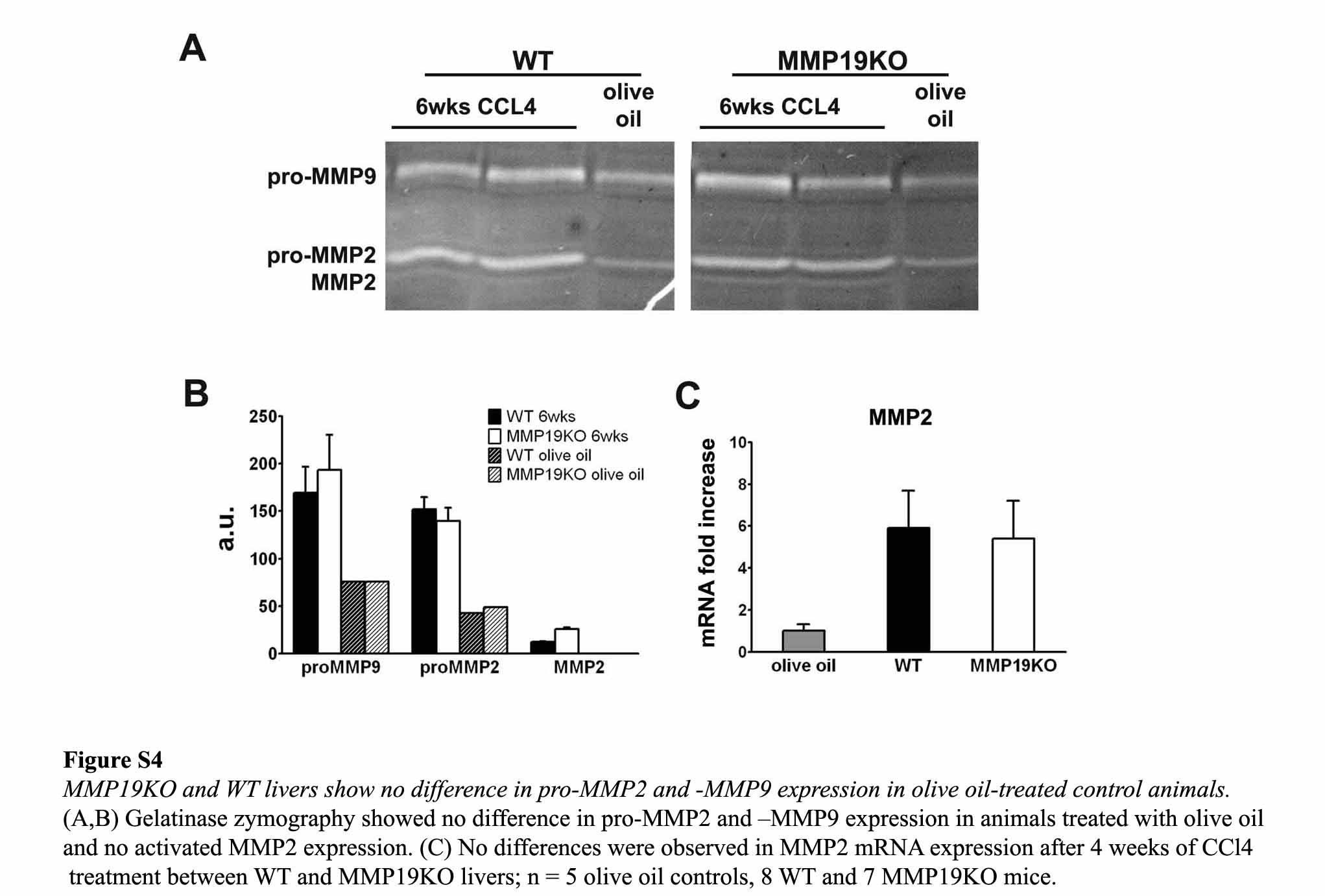

Supplement: Figure S4 — MMP19KO and WT livers show no difference in pro-MMP2 and -MMP9 expression in olive oil-treated control animals. (A,B) Gelatinase zymography showed no difference in pro-MMP2 and -MMP9 expression in animals treated with olive oil and no activated MMP2 form expression. (C) No differences were observed in MMP2 mRNA expression after 4 weeks of CCl4 treatment between WT and MMP19KO livers; n = 5 olive oil controls, 8 WT and 7 MMP19KO mice. (JPG) [file pone.0046271.s005.jpg]

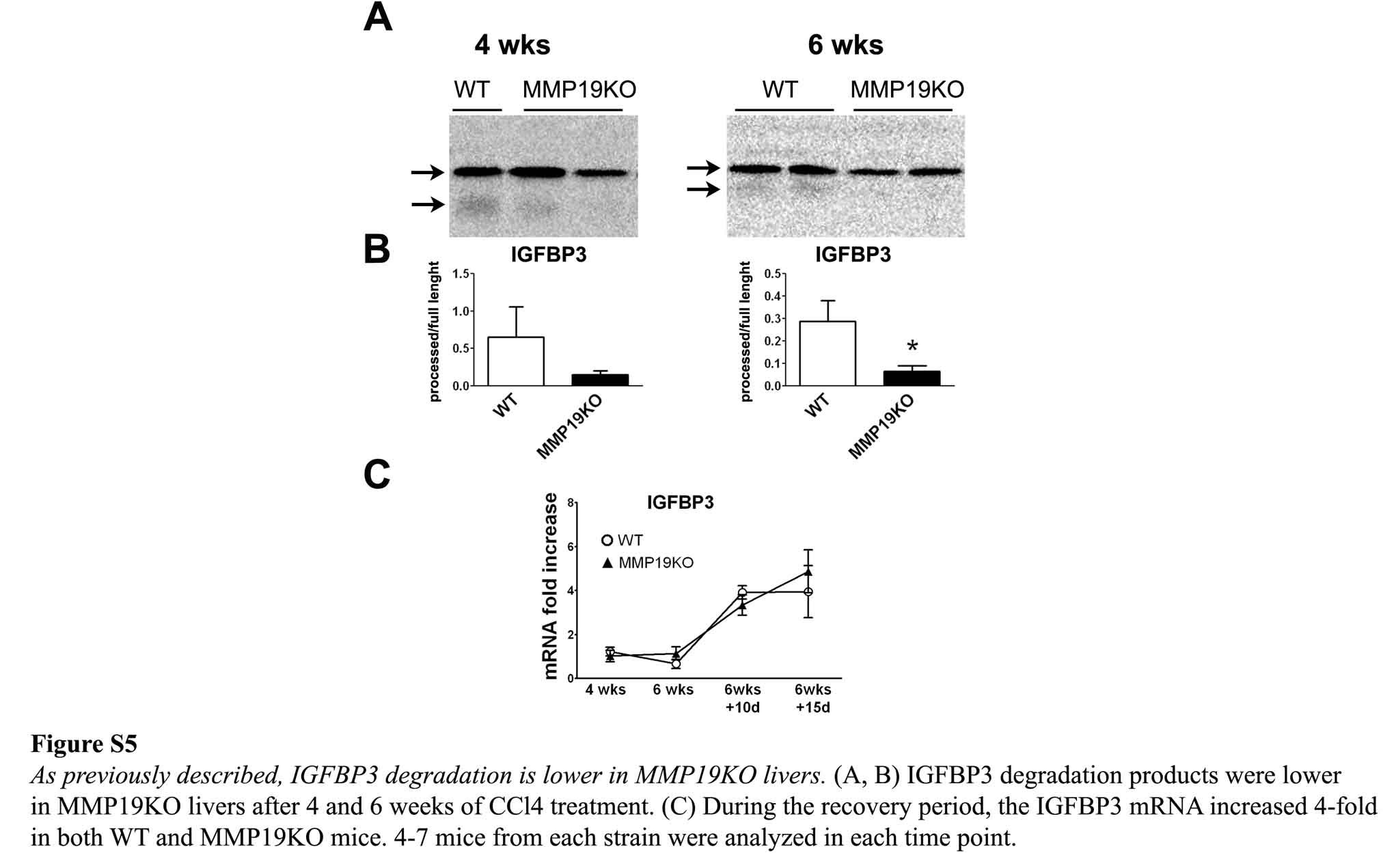

Supplement: Figure S5 — As previously described, IGFBP3 degradation is lower in MMP19KO livers. (A, B) IGFBP3 degradation products were lower in MMP19KO livers after 4 and 6 weeks of CCl4 treatment. (C) During the recovery period, the IGFBP3 mRNA increased 4-fold in both WT and MMP19KO mice. 4–7 mice from each strain were analyzed at each time point. (JPG) [file pone.0046271.s006.jpg]
