# Supplementary material for: Hepatoprotective Effect of MMP-19 Deficiency in a Mouse Model of Chronic Liver Fibrosis
Source: PLoS One. 2012 Oct 9;7(10):e46271. doi: 10.1371/journal.pone.0046271 (PMC3467204; doi:10.1371/journal.pone.0046271)
Supplement: Table S1 — MCP1 and KC levels in MMP19KO and WT mice after 4 or 6-week treatment with CCl4 were measured in serum using an Elisa kit. (DOC) [file pone.0046271.s007.doc]

**Supplemental Table**

MCP1 and KC levels in MMP19KO and WT mice after 4 or 6-week treatment with CCl_4_ were measured in serum using an Elisa kit.

|  | **4 wks** | | **6 wks** | |
| --- | --- | --- | --- | --- |
|  | MCP-1 (pg/ml) | KC (pg/ml) | MCP-1 (pg/ml) | KC (pg/ml) |
| **WT** | 182 ± 70 | 328 ± 83 | 168 ± 24 | 187 ± 14 |
| **MMP19KO** | 136 ± 78 | 365 ± 115 | 101 ± 7 | 141 ± 17 |
| p value (WT vs. MMP19KO) | n.s. | n.s. | 0.01 | n.s. |
